# A cross-sectional study of factors associated with dog ownership in Tanzania

**DOI:** 10.1186/1746-6148-4-5

**Published:** 2008-01-29

**Authors:** Darryn L Knobel, M Karen Laurenson, Rudovick R Kazwala, Lisa A Boden, Sarah Cleaveland

**Affiliations:** 1 Royal (Dick) School of Veterinary Studies, University of Edinburgh, Easter Bush, Roslin, Midlothian, EH25 9RG, UK; 2 Africa Regional Office, Frankfurt Zoological Society, P.O. Box 14935, Arusha, Tanzania; 3 Department of Medicine and Public Health, Faculty of Veterinary Medicine, Sokoine University of Agriculture, P.O. Box 3021, Chuo Kikuu, Morogoro, Tanzania; 4 Centre for Infectious Disease, University of Edinburgh, Ashworth Laboratories, West Mains Road, Edinburgh, EH9 3JT, UK

## Abstract

**Background:**

Mass vaccination of owned domestic dogs is crucial for the control of rabies in sub-Saharan Africa. Knowledge of the proportion of households which own dogs, and of the factors associated with dog ownership, is important for the planning and implementation of rabies awareness and dog vaccination programmes, and for the promotion of responsible dog ownership. This paper reports the results of a cross-sectional study of dog ownership by households in urban and rural communities in the United Republic of Tanzania.

**Results:**

Fourteen percent (202) of 1,471 households surveyed were identified as dog-owning, with an average of 2.4 dogs per dog-owning household. The percentage of dog-owning households was highest in inland rural areas (24%) and lowest in coastal urban communities (7%). The overall human:dog ratio was 14:1. Multivariable logistic regression revealed that households which owned cattle, sheep or goats were much more likely to own dogs than households with no livestock. Muslim households were less likely to own dogs than Christian households, although this effect of religion was not seen among livestock-owning households. Households were more likely to own a dog if the head of the household was male; if they owned a cat; or if they owned poultry. Dog ownership was also broadly associated with larger, wealthier households.

**Conclusion:**

The human:dog ratios in Tanzania are similar to those reported elsewhere in sub-Saharan Africa, although cultural and geographic variation is evident. Estimation of the number of owned dogs, and identification of household predictors of dog ownership, will enable targeted planning of rabies control efforts.

## Background

Domestic dogs are ubiquitously associated with human populations in nearly all parts of the world. Reasons for keeping or tolerating dogs vary across societies and may involve aspects of security, companionship, transport, food acquisition or religious beliefs [1]. Whilst broad cultural patterns in human-dog relationships can be defined [2-4], within these trends there remains considerable variation between individual social units (e.g. families or households) in attitudes towards and associations with dogs [5-8]. Understanding the demographics and predictors of dog ownership at a household level may be of importance in fields such as public health [9-11] or social psychology [12-16], or of commercial interest in the provision and marketing of veterinary services and products [17].

Although many societies derive benefits from their associations with dogs, dogs may also pose significant risks to human health and well-being [18-20]. Of these, the transmission of rabies virus undoubtedly carries the most severe consequences. Dogs are the most important reservoirs of rabies virus in many parts of the world, particularly in developing regions such as sub-Saharan Africa, the Indian subcontinent and south-east Asia [21], and the vast majority of human rabies fatalities (typically >90%) in these regions are as a consequence of exposure to rabid dogs [22]. It is estimated that canine rabies kills over 55,000 people each year in Africa and parts of Asia alone [23]. In addition to human deaths, canine rabies also imposes an economic burden on societies and individuals, in the form of costly post-exposure vaccinations required to prevent the development of clinical rabies following exposure to a suspected rabid animal [23]. The burden of canine rabies often falls disproportionately on those least able to bear it: public health departments in developing countries already faced with controlling burgeoning rates of tuberculosis, malaria and HIV; low-income rural households from which patients may need to travel long distances to seek treatment; and children, who face a higher risk of rabies exposure and death [23].

In areas where canine rabies is endemic, epidemiological theory and economic analysis suggest that control efforts must focus on establishing and maintaining a high (>70%) vaccination coverage in the dog population [24,25]. Understanding the factors that affect the accessibility of dogs for vaccination is thus critical to local and national rabies control programmes. In sub-Saharan Africa, the results of several recent studies suggest that the proportion of unowned, feral dogs is low: the majority of dogs are accessible for vaccination through households which claim responsibility for them [26,27]. Given this, a deeper understanding of the household-level factors associated with dog ownership in these communities may be important for public health planning of rabies awareness programmes and dog vaccination campaigns. While a number of studies have explored the factors associated with dog ownership among communities in developed countries [8,28-31], few equivalent studies have been undertaken in developing economies. This paper reports the results of a large-scale cross-sectional study of dog ownership patterns across 12 study sites, encompassing both urban and rural agro-pastoralist households in central and eastern Tanzania.

## Results

### Dog ownership patterns

A total of 1,471 households were interviewed between October 2004 and July 2005 (9.7% of 15,220 identified households across 12 study sites), of which 202 (13.7%) were dog-owning households (DOHH). Within the sample of DOHH, 81 households (40%) owned one dog, 72 (36%) two dogs, 13 (6%) three dogs, and the remainder owned between 4–12 dogs each (mean number of dogs per DOHH 2.38 95% CI 2.34–2.42). A breakdown of the dog ownership patterns by study site is given in Table 1. Of the 1,072 non-dog-owning households (NOHH) who gave a reason for non-ownership, 29.2% said they disliked dogs, 26.4% felt they lacked the time and/or space to take care of a dog, 17.4% did not feel the necessity of having a dog, 10.9% had not yet replaced a previous dog that had died or disappeared, 8.6% stated that it was against their cultural or religious views and 7.5% felt that dogs were too expensive to acquire and look after.

**Table 1:** Patterns of dog ownership across 12 study sites in Tanzania. DOHH = Dog-owning households. Figures in bold are mean and 95% confidence intervals (in parentheses) derived from 10,000 bootstrap samples of the original data

	Urban						Rural					
		
	Inland			Coastal			Inland			Coastal		
				
Study site	Koro	Maje	Mbuy	Dund	Ngam	Teme	Bwak	Chin	Kiri	Kira	Kiro	Kisa
% DOHH	16.8	14.1	14.7	10.3	8.1	2.5	22.0	12.7	33.8	18.1	11.5	19.4
	**15.1 (14.1–16.0)**			**7.1 (6.5–7.8)**			**23.9 (22.1–25.8)**			**16.4 (14.8–18.1)**		
	**11.5 (10.9–12.1)**						**20.1 (18.9–21.4)**					
	**13.7 (13.2–14.3)**											
Households per dog	2.7	2.7	3.6	3.1	8.9	32.6	1.2	3.9	1.4	2.3	3.1	2.3
	**3.0 (2.7–3.2)**			**5.8 (5.1–6.7)**			**1.6 (1.5–1.8)**			**2.5 (2.2–2.8)**		
	**3.8 (3.6–4.1)**						**2.0 (1.8–2.1)**					
	**3.1 (2.9–3.2)**											
Humans per dog	15.4	8.3	11.1	11.0	78.6	214.6	6.6	17.5	5.3	8.3	11.7	14.0
	**14.4 (13.2–15.7)**			**27.2 (23.9–31.1)**			**7.6 (6.8–8.6)**			**10.8 (9.5–12.3)**		
	**18.0 (16.8–19.4)**						**8.9 (8.2–9.7)**					
	**14.3 (13.5–15.1)**											

### Factors affecting dog ownership

The results of the univariable analysis of the effects of both the original and combined variables on dog ownership status are presented in Table 2. The household socio-demographic types identified using hierarchical cluster analysis (see Methods) are summarised in Table 3. In the final multivariable mixed-effect model (Table 4), dog ownership was significantly associated with the gender composition of the adult members of the household, household socio-demographic type, possession of poultry, cat ownership, religion of the head of the household and livestock ownership. The odds of dog ownership were greater in male-led households when compared to households in which no adult male was present (OR 3.5, 95% CI 1.7–7.2). Compared to household socio-demographic type 1, the odds of dog ownership were greater in type 4 households (better educated, wealthier, larger households; OR 2.8 95% CI 1.5–5.0) and type 5 households (moderately wealthier, larger households, older household heads; OR 3.1 95% CI 1.8–5.3). Cat-owning households had greater odds of dog ownership (OR 2.0 95% CI 1.2–3.3), as did households which kept poultry (OR 2.0 95% CI 1.4–2.9), when compared to non-cat and -poultry owning households, respectively. One interaction term, between livestock ownership and religion, was significant in the final model. Among non-livestock-owning households, Muslim households had lower odds of dog ownership than Christian households (OR 0.3 95% CI 0.2–0.4); however, among livestock-owning households there was no effect of religion on dog ownership (OR 0.9 95% CI 0.4–1.9). Livestock ownership increased the log odds of dog ownership by 0.8 in Christian households (OR 2.1 95% CI 1.2–3.7) and by 1.9 in Muslim households (OR 6.4 95% CI 3.1–13.1).

**Table 2:** Univariable analysis of factors associated with dog ownership in Tanzania

**Variable**	**Total (n = 1471)**	**DOHH (%) (n = 202)**	**NOHH (%) (n = 1269)**	**Coefficient**	**Standard error**	*p*-value*	**Odds ratio (OR)**	**95% confidence interval (CI)**
**Sex of head of household**								
Female	613	47 (23)	566 (45)				1(REF)	
Male	855	155 (77)	700 (55)	0.98	0.176	**< 0.001**	2.67	1.89–3.76
Missing	3	0	3					
**Age of head of household (years)**						**< 0.001**		
<25	150	11 (5)	139 (11)				1(REF)	
25–34	352	30 (15)	322 (25)	0.16	0.367	0.65	1.18	0.57–2.42
35–44	353	41 (20)	312 (25)	0.51	0.355	0.15	1.66	0.83–3.33
45–54	266	56 (28)	210 (17)	1.21	0.348	<0.001	3.37	1.71–6.66
55–64	167	27 (13)	140 (11)	0.89	0.377	0.02	2.44	1.16–5.10
65–74	99	21 (10)	78 (6)	1.22	0.398	0.002	3.40	1.56–7.43
75+	77	15 (7)	62 (5)	1.12	0.425	0.009	3.06	1.33–7.04
Missing	7	1	6					
**Occupation of head of household**								
Elementary	794	114 (58)	680 (54)				1(REF)	
Secondary	660	84 (42)	576 (46)	-0.14	0.155	**0.37**	0.87	0.64–1.18
Missing	17	4	13					
**Level of education of head of household**								
None	184	26 (13)	158 (13)				1(REF)	
Primary	927	101 (51)	826 (65)	-0.30	0.236	0.21	0.74	0.47–1.18
Secondary	232	43 (22)	189 (15)	0.32	0.271	0.23	1.38	0.81–2.35
Tertiary	118	27 (14)	91 (7)	0.59	0.305	0.05	1.80	0.99–3.28
Missing	10	5	5					
**Religion of head of household**								
Christian	598	127 (66)	471 (37)				1(REF)	
Muslim	852	66 (34)	786 (63)	-1.16	0.163	**<0.001**	0.31	0.23–0.43
Missing	21	9	12					
**Number of household occupants**						**< 0.001**		
1	89	4 (2)	85 (7)				1(REF)	
2	135	10 (5)	125 (10)	0.53	0.608	0.38	1.70	0.52–5.60
3	247	20 (10)	227 (18)	0.63	0.562	0.26	1.87	0.62–5.64
4	315	40 (20)	275 (22)	1.13	0.539	0.04	3.09	1.08–8.89
5	217	28 (14)	189 (15)	1.15	0.550	0.04	3.15	1.07–9.26
6	175	26 (13)	149 (12)	1.31	0.554	0.02	3.71	1.25–10.98
7+	288	74 (37)	214 (17)	1.99	0.526	<0.001	7.35	2.62–20.60
Missing	5	0	5					
**Household asset quintile**						**< 0.001**		
1	277	44 (22)	233 (19)				1(REF)	
2	290	23 (12)	267 (21)	-0.78	0.273	0.004	0.46	0.27–0.78
3	292	35 (18)	257 (21)	-0.33	0.244	0.18	0.72	0.45–1.16
4	296	27 (14)	269 (22)	-0.63	0.260	0.02	0.53	0.32–0.89
5	290	71 (36)	219 (18)	0.54	0.214	0.01	1.72	1.13–2.61
Missing	26	2	24					
**How long in area?**								
≥1 year	1346	187 (97)	1159 (94)				1(REF)	
<1 year	75	6 (3)	69 (6)	-0.62	0.433	**0.12**	0.54	0.23–1.26
Missing	50	9	41					
**Is the respondent the head of household?**								
No	350	83 (41)	267 (21)				1(REF)	
Yes	1121	119 (59)	1002 (79)	-0.96	0.159	**<0.001**	0.38	0.28–0.52
**Presence of a male adult occupant**								
No	234	10 (5)	224 (18)				1(REF)	
Yes	1232	192 (95)	1040 (82)	1.42	0.333	**<0.001**	4.14	2.15–7.94
Missing	5	0	5					
**Presence of a female adult occupant**								
No	98	11 (5)	87 (7)				1(REF)	
Yes	1368	191 (95)	1177 (93)	0.25	0.329	**0.44**	1.28	0.67–2.45
Missing	5	0	5					
**Presence of children (<16 years)**								
No	357	41 (20)	316 (25)				1(REF)	
Yes	1109	161 (80)	948 (75)	0.27	0.187	**0.14**	1.31	0.91–1.89
Missing	5	0	5					
**Presence of a male child occupant**								
No	639	69 (34)	570 (45)				1(REF)	
Yes	827	133 (66)	694 (55)	0.46	0.159	**0.003**	1.58	1.16–2.16
Missing	5	0	5					
**Presence of a female child occupant**								
No	690	88 (44)	602 (48)				1(REF)	
Yes	776	114 (56)	662 (52)	0.16	0.153	**0.28**	1.18	0.87–1.59
Missing	5	0	5					
**Ownership of cattle**								
No	1383	157 (78)	1226 (97)				1(REF)	
Yes	86	45 (22)	41 (3)	2.15	0.232	**<0.001**	8.57	5.44–13.50
Missing	2	0	2					
**Ownership of small stock (sheep/goats)**								
No	1358	159 (79)	1199 (95)				1(REF)	
Yes	111	43 (21)	68 (5)	1.56	0.212	**<0.001**	4.77	3.15–7.23
Missing	2	0	2					
**Ownership of poultry**								
No	1041	95 (47)	946 (75)				1(REF)	
Yes	428	107 (53)	321 (25)	1.20	0.155	**<0.001**	3.32	2.45–4.50
Missing	2	0	2					
**Ownership of pigs**								
No	1455	196 (97)	1259 (99)				1(REF)	
Yes	14	6 (3)	8 (1)	1.57	0.545	**0.007**	4.82	1.65–14.03
Missing	2	0	2					
**Ownership of cat/s**								
No	1349	171 (85)	1178 (93)				1(REF)	
Yes	120	31 (15)	89 (7)	0.88	0.224	**<0.001**	2.40	1.55–3.72
Missing	2	0	2					
**Ownership of other animals**								
No	1432	189 (94)	1243 (98)				1(REF)	
Yes	37	13 (6)	24 (2)	1.27	0.353	**<0.001**	3.56	1.78–7.12
Missing	2	0	2					
**COMPOSITE VARIABLES:**								
**Adult gender composition**						**< 0.001**		
No adult male occupant	225	9 (4)	216 (17)				1(REF)	
Female head; adult male occupant/s	384	38 (19)	346 (27)	0.97	0.381	0.01	2.64	1.25–5.56
Male head	855	155 (77)	700 (55)	1.67	0.352	<0.001	5.31	2.67–10.58
Missing	7	0	7					
**Household socio-demographic type**						**< 0.001**		
1	365	28 (14)	337 (27)				1(REF)	
2	297	30 (15)	267 (21)	0.30	0.275	0.27	1.35	0.79–2.32
3	349	44 (22)	305 (24)	0.55	0.254	0.03	1.74	1.05–2.86
4	213	39 (19)	174 (14)	0.99	0.265	<0.001	2.70	1.61–4.53
5	247	61 (30)	186 (15)	1.37	0.246	<0.001	3.95	2.44–6.39
**Composition of child occupants**						**0.014**		
Both sexes	494	86 (43)	408 (32)				1(REF)	
Female only	282	28 (14)	254 (20)	-0.65	0.232	0.005	0.52	0.33–0.82
Male only	333	47 (23)	286 (23)	-0.25	0.197	0.21	0.78	0.53–1.15
No children	357	41 (20)	316 (25)	-0.49	0.204	0.02	0.62	0.41–0.92
Missing	5	0	5					
**Ownership of livestock (cattle/sheep/goats)**								
No	1321	140 (69)	1181 (93)				1(REF)	
Yes	148	62 (31)	86 (7)	1.81	0.189	**<0.001**	6.08	4.20–8.81
Missing	2	0	2					

*Bolded *p*-values are likelihood ratio test *p*-values and unbolded *p*-values are Wald test *p*-values.

**Table 3:** Household socio-demographic types derived from hierarchical cluster analysis of age and level of education of the head of the household, number of occupants in the household (household size) and asset quintile score (AQ, with 1 = poorest and 5 = least poor)

**Type 1**
Less well educated (21% none, 67% primary school only); less wealthy (92% AQ1–3, with 31% AQ1); smaller households (80% <4 occupants); younger household heads (80% <55 years, with 47% <35 years)
**Type 2**
Moderately educated (40% secondary/tertiary education, 58% primary school only); wealthy (94% AQ4–5, with 44% AQ5); smaller households (all <5 occupants, with 50% having only 2–3 occupants); younger household heads (60% 25–44 years)
**Type 3**
Less well educated (10% none, 80% primary school only); less wealthy (all AQ1–3, with 39% AQ1); larger households (all 4+ occupants, with 27% 7+); younger household heads (all <65 years, with 35% <35 years)
**Type 4**
Better educated (50% secondary/tertiary education, 49% primary only); wealthy (95% AQ4–5, with 50% AQ5); larger households (all 5+ occupants, with 33% 7+); younger household heads (all <55 years, with 30% <35 years)
**Type 5**
Less well educated (25% none, 54% primary school only); moderately wealthy (50% AQ3–4); larger households (all 4+ occupants, with 50% 7+); older household heads (all 45+ years)

**Table 4:** Multivariable mixed-effects logistic regression model of factors associated with dog ownership in Tanzania, with study site as a random effect (n = 1,471)

**Variable**	**Coefficient**	**Standard error**	*p*-value*	**Odds ratio (OR)**	**95% confidence interval**
**Adult gender composition**			**< 0.001**		
No adult male occupant				1 (REF)	
Female head; adult male occupant/s	0.64	0.402	0.12	1.90	0.87–4.18
Male head	1.26	0.366	<0.001	3.52	1.72–7.20
**Household socio-demographic type**			**< 0.001**		
Type 1				1 (REF)	
Type 2	0.38	0.311	0.22	1.46	0.79–2.69
Type 3	0.21	0.279	0.45	1.24	0.72–2.13
Type 4	1.01	0.302	<0.001	2.75	1.52–4.98
Type 5	1.12	0.278	<0.001	3.08	1.78–5.32
**Ownership of poultry**			**< 0.001**		
No				1 (REF)	
Yes	0.69	0.194	< 0.001	1.99	1.36–2.91
**Ownership of cat/s**			**0.01**		
No				1 (REF)	
Yes	0.68	0.265	0.01	1.97	1.18–3.32
**Ownership of livestock**					
No				1 (REF)	
Yes	0.75	0.417	0.01	2.11	1.20–3.73
**Religion of head of household**					
Christian				1 (REF)	
Muslim	-1.23	0.208	< 0.001	0.29	0.19–0.44
**Interaction between livestock ownership and religion**	1.11	0.445	**0.02**	3.02	1.26–7.23
**Intercept**	-3.38	0.417			

*Bolded *p*-values are likelihood ratio test *p*-values and unbolded *p*-values are Wald test *p*-values.

Adding study site as a random effect in a generalised linear mixed-effects model containing the selected variables and interaction term as fixed effects resulted in minimal changes (<9%) to the coefficients and standard errors compared to the single-level model. Although there was no evidence to reject the null hypothesis of no random variation between study sites (likelihood-ratio test *P *= 0.07, d.f. = 1, one-sided alternative hypothesis; [32]), the random effect of study site was retained in the final model in accordance with the hierarchical nature of the study design [33]. Table 4 presents the results of this mixed-effects model.

The fixed-effects multivariable model appeared to fit the data adequately (Hosmer-Lemeshow goodness-of-fit test statistic = 9.99, d.f. = 8, *P *= 0.29). The area under the ROC curve was 0.80, indicating the model has good predictive ability. Four influential covariate patterns were identified. Removal from the model of households within two of these covariate patterns had minimal impact on the estimated coefficients (<15%). The two remaining covariate patterns had large values of delta beta and delta χ^2 ^(measures of the effect of covariate patterns on regression coefficients and fit of the model, respectively).

## Discussion

This paper presents the results of the first study of patterns and predictors of dog ownership in Tanzania. The numbers of owned dogs are similar to those reported elsewhere in sub-Saharan Africa [9,34-38], with the point estimates for human:dog ratios falling within the 95% confidence intervals of estimates from a meta-analysis of data from both urban and rural settings in Africa [23]. The overall proportion of dog-owning households identified in the study sample (13.7%) is lower than reported national estimates from non-African countries including the United States (36.1%, [39]), Japan (24.2%, [40]), Taiwan (22.9%, [5]), and Sweden (15.5%, [41]). However, the mean number of dogs per dog-owning household (2.2) is higher, with a lower proportion of dog-owning households only keeping a single dog (40%). Corresponding figures for other countries are: United States 1.7 & 63%, Taiwan 1.6 & 69.5%, Sweden 1.4 & 77.9%. The variation seen in the proportion of households owning dogs, with rural areas having a high proportion of dog-owning households and coastal urban areas in particular displaying the inverse, reflects the geographic heterogeneity in the distribution of factors operating at a household level: livestock keeping is more common in rural than in urban areas (69.7% of rural households in this study engaged in some form of livestock keeping, compared to 18.2% of urban households), and a higher proportion of Tanzania's coastal population are Muslim (77.6% vs. 40.8% in inland areas – data from this study).

Several other studies have reported an association between the adult gender composition of a household and dog ownership. In contrast to the present study, Westgarth et al. [8] found that it was the presence of an adult female in the household that was positively associated with ownership among a semi-rural population in the UK. Similarly, a study of young, single-member households in the U.S.A determined that females were more likely to own dogs [42]. These results reflect the generally higher degree of attachment to companion animals exhibited by females in several North American and European studies [6,7,43-45]. It has been shown that the sex of the head of the household is highly correlated with several other factors which may predict dog ownership. Katapa [46] found that female-headed households in Tanzania were more likely to have children under the age of five, fewer than six occupants, fewer adult males, no radio (a common household asset in the current study) and to be generally poorer than male-headed households. Although similar associations were seen when testing for collinearity among independent variables in the current study, these were accounted for in the model-building process, suggesting that there is an inherent association between male-led households and dog ownership. This view is supported by the results of a related study assessing owners' attitudes towards dogs, based on the same population sample as the current study, which showed that male respondents had a significantly more positive attitude towards dogs than females (Knobel et al. in prep). Other studies in non-Western countries have found a similar relationship. In a random telephone survey in Taiwan, Hsu et al. [5] found that male respondents were more likely to report having ever owned a dog than females, and in Kuwait, Al-Fayez et al. [47] reported that males had a more positive attitude towards companion animals, as measured on the Pet Attitude Scale [48].

Although multicollinearity between independent variables can give rise to spurious results [49], the problem has largely been ignored in the few studies examining the predictors of dog ownership among households (but see [8]). The high degree of correlation between independent variables in the current study necessitated the formation of new, composite variables which make interpretation and comparison of results to previous studies less straightforward. Nevertheless, broad patterns can be identified. Socio-demographic type 4 and 5 households were more likely to own dogs than other household types. Type 4 households were almost exclusively urban households (99%). When compared to the other major category of urban households, type 2 (99% of all type 2 households were also urban), the major distinction is in household size, with type 4 households all having five or more occupants (Table 3). Type 5 households also tended to be larger households, with half having seven or more occupants. This association between household size and dog ownership has been consistently reported across studies [5,8,29,31,50-52], although only the latter two studies employed a multivariable approach. In the univariable analysis in the current study, the number of occupants in a household was strongly associated with the household's asset quintile, the age of its head occupant and the likelihood of livestock ownership. Larger households usually consist of several related family units, the oldest (usually male) member of which tends to be deferred to as the household head. Because asset score and livestock ownership are measured at a household level, rather than on a *per capita *basis, it is also unsurprising that larger households would rank higher in these aspects. Despite these complex interrelationships, the hierarchical cluster analysis does suggest an effect of household size *per se *on dog ownership. It also suggests that dog-owning households tend to have higher asset quintile scores (with the exception of type 2 households: smaller, wealthy urban households). This association between dog-ownership and a proxy measure of household income is a finding common to a number of American studies [29,31,50-52]. However, this does not extend to an association with occupation classification, which was shown to be non-significant in the current study and in multivariable analyses by both Leslie et al. [30] and Westgarth et al. [8]. The latter authors concluded that the effects of occupation or income are likely to be intertwined with other household characteristics, primarily life stage grouping [42]. This conclusion is supported by the hierarchical cluster analysis of household types presented here, and highlights the importance of examining and reporting unconditional associations between variables after multivariable analysis.

While the association demonstrated between dog- and livestock-ownership fits with the cultural-materialist view of pet keeping commonly invoked to explain the phenomenon in non-Western societies [53], wherein it is assumed that pets are kept by a society solely if doing so results in a net increase to the efficiency of that society's production system, the interpretation of this finding may not be straightforward. In a survey of dog-owning households in the 12 study sites, the authors found that only 12.7% (75/591) of livestock-owning households gave as their primary reason for owning dogs "To protect livestock against predators". The majority (61.9%) reported keeping dogs "To guard the household against human intruders", as did the majority (76.4%) of non-livestock-owning households. A higher proportion of livestock owners did report keeping dogs to chase pests away from crops (23.5% vs. 15.2% of non-livestock-owning households), reflecting the largely mixed agro-pastoralist production systems in the non-urban areas of this study. While these findings by no means refute a cultural-materialist viewpoint, they do suggest that other factors, possibly associated with broader constructs related to rural lifestyles in general (e.g. attitudes towards animals) rather than livestock keeping alone, may be in operation.

Whatever the association between livestock and dog ownership, it seems strong enough to override pre-existing cultural aversions to dogs. In Islam, dogs are traditionally considered impure, although dog ownership itself is not proscribed [54]. This is reflected in the significantly lower odds of dog ownership among Muslim households, compared to their Christian counterparts. However, among livestock-owning households this distinction no longer holds, with respondents of either religion being equally likely to own dogs. Livestock ownership greatly increases the odds of a Muslim household owning dogs. This does provide evidence for a utilitarian role for dogs among livestock owners, the benefits of which outweigh any cultural considerations.

Regression diagnostics revealed two influential covariate patterns, with large negative Pearson residuals, suggesting that a higher proportion of households with these covariate patterns were predicted to be dog-owning than was the case. Re-examination of the data showed that 50% (10/20) of non-owning households with these two covariate patterns gave as their reason for non-ownership, "Previous dogs died/disappeared and have not yet been replaced", compared to just 10% of households within other covariate patterns. There is thus an inclination towards dog ownership within these households, supporting the predictive ability of the model.

## Conclusion

It is hoped that the results presented in this paper may assist veterinary and public health officials in the planning and implementation of rabies control programmes in Tanzania and elsewhere. The consistent results of human:dog ratios across studies, together with the identification of large-scale proxies for household-level predictors (primarily urban/rural and coastal/inland) suggest that crude estimates of dog numbers could be obtained by extrapolation from human population figures through application of the relevant ratio. This will allow more accurate estimation and targeting of resources for national-level rabies control programmes, and may assist with the prediction of potential rabies 'hot-spots' or outbreak zones. Identification and understanding of household-level predictors of dog ownership will also permit the focused implementation of education and awareness campaigns that will be necessary to ensure the success of a national control strategy.

## Methods

### Study design

Study sites were selected from each of three coastal (Dar es Salaam, Tanga and Pwani) and three inland regions (Kilimanjaro, Dodoma and Morogoro) in the United Republic of Tanzania. Within each region, the district containing the regional capital or major coastal city was selected, and from these a single urban ward was randomly selected. If the selected district contained a mix of urban and rural wards, then a single rural ward was also selected at random from the same district. If however the selected district was largely urban, then a rural ward was randomly selected from an adjacent rural district. Within selected wards, study site sizes were standardized such that urban sites contained 1,000–2,000 households, and rural sites 500–1,000. If the selected ward was too large, an appropriate number of contiguous administrative units (sub-wards) were selected as the study site. In this way, three study sites were selected in each of the four possible combinations of urban/rural and inland/coastal categories. These categories were chosen as they were believed to reflect hypothesised differences in socio-cultural aspects of dog ownership, such as the economic status and religious beliefs of households.

Within each study site, a dog rabies vaccination campaign was organised as part of a larger research project. Prior to the vaccination campaign, a simple random sample of households within a study site was selected for the cross-sectional survey. The working definition of a "household" was derived from the 2002 Tanzania Population and Housing Census [55]. If a sampling frame of all households was available from administrative authorities, a random-number generator was used to obtain a sample of 10% of the households, identified by the name of chief occupant. If no sampling frame was available, interviewers visited all households in the study area and determined if an interview should be conducted by blindly selecting coloured markers (one red and nine black). If the red marker was drawn the household was selected for an interview. If no adult (≥ 16 years) was present, or if occupants declined to be interviewed, the next household (on the sampling frame list where applicable; physically adjacent where not) was selected; however, very few households were excluded in this fashion (<5%). A standardised questionnaire was administered to the most senior adult occupant of the household present, by one of four trained interviewers. Individual-level factors such as sex, age, level of education, occupation and religion were recorded for the head of the household. An asset approach was used as a measure of socio-economic status [56]: the questionnaire included details of the construction material of the housing unit, ownership of certain consumer items (working radio, television, refrigerator, bicycle, motorcycle, car) and availability of utilities such as electricity and piped water. Principal components analysis was used to assign a weight to each household asset and the resulting asset scores were standardized in relation to a normal distribution with a mean of zero and a standard deviation of one [57]. Standardized scores were summed for each households, and households classified according to asset quintile, with 1 = poorest and 5 = least poor.

### Model building

Households were classified as dog-owning (DOHH) or non-dog-owning (NOHH). The relationships between potential predictors and dog ownership status were examined using univariable logistic regression. Factors with a likelihood-ratio test *p *value of ≤0.25 were considered for entry into a multivariable logistic regression model. Prior to this, multicollinearity between these selected variables was assessed using Pearson's Chi-squared or Fisher's exact tests, and through examination of the generalized variance inflation factors when all variables were entered into a preliminary main effects logistic regression model. Variables exhibiting high collinearity were either combined to form new variables, or were excluded from the model. This decision was based upon assessment of the potential functional relationships between the variables and consideration of causal effects of dog ownership. Four household socio-demographic variables (level of education and age of head of household, asset quintile and number of occupants) were found to be highly collinear. Hierarchical cluster analysis using Ward's minimum variance method was used to group these into a single new variable, household socio-demographic type, with five levels. Two variables relating to gender (sex of head of household and presence of an adult male occupant) were combined, as were the variables relating to the presence and sex of children in the household. Ownership of cattle and/or small stock (sheep and goats) was combined into a single dichotomous variable, livestock ownership. One variable (whether the respondent was the head of the household or not) was excluded from the model. This variable was found to be highly collinear with a number of independent variables, but was considered to be causally unrelated to dog ownership.

A multivariable logistic regression model of dog ownership was constructed by backward stepwise selection of variables. Variables were retained in the model if the likelihood-ratio test *p *values were <0.05. The Wald test *p*-value was used when comparing categories with the reference category. The potential confounding effects of those variables not retained in the final model were assessed by refitting each variable in succession into the final model and inspecting the percentage change in the odds ratios of the retained variables. A variable was deemed a confounder if it resulted in >20% change in the odds ratio [33]. All two-way interaction terms between variables in the final main effects model were assessed, again using backward stepwise selection. Finally, the effect of the study design was taken into account by entering study site as a random effect in the model and examining the impact on the coefficients estimated in the single-level model. Statistical analysis was done using R version 2.4.1 (The R Foundation for Statistical Computing [58])

### Fit of the model and regression diagnostics

The fit of the final fixed-effects model was assessed using the Hosmer-Lemeshow goodness-of-fit test [59], and its predictive ability determined by generating a receiver operator characteristic (ROC) curve. Regression diagnostics were performed on the model to identify covariate patterns with the greatest leverage, delta betas, delta χ^2 ^and delta deviance values (measures of the effect of covariate patterns on regression coefficients and fit of the model). Households with these covariate patterns were then removed from the model and the change in the value of the coefficients was examined [59].

## Authors' contributions

DLK participated in the design of the study, carried out the data collection, performed the data analysis and drafted the manuscript. MKL and SC conceived of and raised funding for the study, and together with RRK participated in its design and co-ordination. LAB assisted with data analysis and drafting the manuscript. All authors read and approved the final manuscript.
